# Sequence-based GWAS reveals genes and variants associated with predicted methane emissions in French dairy cows

**DOI:** 10.1186/s12711-025-00977-z

**Published:** 2025-06-17

**Authors:** Solène Fresco, Marie-Pierre Sanchez, Didier Boichard, Sébastien Fritz, Pauline Martin

**Affiliations:** 1 Eliance, 149 Rue de Bercy, 75595 Paris cedex 12, France; 2 Université Paris-Saclay, INRAE, AgroParisTech, GABI, 78350 Jouy-en-Josas, France

## Abstract

**Background:**

Due to their contribution to global warming, methane emissions from ruminants have been the subject of considerable scientific interest. It has been proposed that such emissions might be reduced using genetic selection; proposed phenotypes differ in the measurement methods used (direct or predicted methane emissions) and in the unit under consideration (g/d, g/kg of milk, g/kg of intake, residual methane emissions). Identifying the quantitative trait loci (QTLs) and candidate genes responsible for genetic variation in methane emissions allows a better understanding of the underlying genetic architecture of these phenotypes. Therefore, the aim of this study was to identify the genomic regions associated with six methane traits predicted from milk mid-infrared (MIR) spectra (0.33 ≤ R^2^ ≤ 0.88) in French Holstein dairy cows using genome-wide association studies at the whole-genome-sequence level.

**Results:**

Six methane emission traits—in g/d, in g/kg of fat- and protein-corrected milk, and in g/kg of dry matter intake—were predicted from milk MIR spectra routinely collected by French milk recording companies. A genome-wide association study of the predicted methane emissions of 40,609 primiparous Holstein cows was conducted using imputed whole-genome-sequence data. This analysis revealed 57 genomic regions of interest; between 1 and 8 QTLs were identified on each of the autosomes except 4, 12, 21, 24 and 26. We identified multiple genomic regions that were shared by two or more predicted methane traits, illustrating their common genetic basis. Functional annotation revealed potential candidate genes, in particular *FASN*, *DGAT1*, *ACSS2*, and *KCNIP4*, which could be involved in biological pathways possibly related to methane production.

**Conclusions:**

The methane traits studied here, which were predicted from milk MIR spectra, appear to be highly polygenic. Several genomic regions associated with these traits contain candidate genes previously associated with milk traits. Functional annotation and comparisons with studies using direct methane measurements support some potential candidate genes involved in biological pathways related to methane production. However, the overlap with genes influencing milk traits highlights the challenge of distinguishing whether these regions genuinely influence methane emissions or reflect the use of milk MIR spectra to predict the phenotypes.

## Background

The contribution of the agricultural sector, particularly cattle breeding, to climate change has been extensively highlighted in recent years. Due to the enteric fermentation in their digestive process, cattle are the primary source of agricultural methane (CH_4_) emissions [1, 2]. Various strategies have been identified to mitigate these emissions, including modifications in feeding strategies, feeding additives, herd management, and genetic selection [3, 4]. Among these proposals, though, only genetic selection offers a cumulative and permanent solution for reducing CH_4_ emissions.

Methane production in ruminants results from the activity of microorganisms within the rumen. The fermentation of ingested feed by rumen bacteria produces hydrogen, which is primarily converted into CH₄ by archaea through methanogenesis [4]. Numerous studies have analyzed the genetic determinism of CH_4_ emissions in cattle [5–9], but the ways in which this process is influenced by host genetics are still unclear and require further research.

One effective approach for investigating the genetic basis of CH_4_ emissions is the use of Genome-Wide Association Studies (GWAS). GWAS identifies genomic markers associated with a phenotype of interest, and significant markers located in the same region collectively indicate Quantitative Trait Loci (QTLs). Within QTL regions, it is possible to identify candidate genes that can improve our understanding of the biological mechanisms underlying CH_4_ emissions. By combining information on both genetic markers and functional annotation, likely causal variants can be proposed. This biological information can be used to design more precise and effective breeding strategies, ultimately contributing to a more sustainable agricultural sector.

Previous GWASs performed on direct CH_4_ measurements identified several genomic regions that were associated with CH_4_ emissions [10–14]. However, the use of direct CH_4_ phenotypes, which are recorded with low-to-medium throughput methods such as Sniffer, GreenFeed, the SF_6_ tracer gas method, and respiration chambers, limits the number of animals that can be included in the analyses and therefore both the detection power and resolution of the results. Some studies have used CH_4_ predicted from feed intake phenotypes [15–17], though these phenotypes are similarly challenging to record at large scale. However, the development of equations to predict CH_4_ emissions from milk mid-infrared (MIR) spectra [18–21] (0.38 ≤ R^2^ ≤ 0.88) has made possible the collection of much larger datasets, enabling more accurate genetic and genomic analyses.

The present study had two primary objectives. Using a large database of French Holstein primiparous cows and milk MIR spectra, which are routinely collected by French milk recording companies, we aimed to (1) detect QTLs associated with CH_4_ emissions predicted from milk MIR spectra, and (2) identify positional candidate genes within those QTLs.

## Materials and methods

### Animals and spectra data

The initial dataset consisted of 1,221,705 milk MIR spectra collected between 70 and 200 days in milk (DIM) from 359,856 primiparous Holstein cows. The following selection criteria were applied. Only lactations from the A-type recording protocol with samples from both morning and evening milkings were kept. The selected lactations started between 2015 and 2021, and each lactation had at least two records. The CH_4_ phenotypes were predicted according to Fresco et al. [20], Vanlierde et al. [19], and Chilliard et al. [18] and are described in the next paragraph. All other information was extracted from the bovine national database (INRAE, CTIG Jouy-en-Josas, France).

### Methane phenotypes

Methane traits were predicted from milk MIR spectra that were standardized according to Grelet et al. [22]. Different prediction equations were applied to the milk MIR spectra to obtain six distinct CH_4_-related traits:

- MeP_FA (in g/d), obtained from the equation (R^2^ = 0.88) developed by Chilliard et al. [18] based on fatty acid composition predicted from milk MIR spectra and CH_4_ measurements with the SF_6_ tracer gas method in a 4 × 4 Latin square experiment.
- MeP_RC (in g/d), obtained from the equation (R^2^ = 0,60) of Vanlierde et al. [19], which was developed using milk MIR spectra and CH_4_ measurements with respiration chambers and the SF_6_ tracer gas method for 299 cows from 7 breeds.
- MeP_direct (in g/d, R^2^ = 0.38), MeY (in g/kg of dry matter intake (DMI), R^2^ = 0.33), and MeI (g/kg of fat- and protein-corrected milk (FPCM), R^2^ = 0.47), obtained from the equations developed by Fresco et al. [20] from milk MIR spectra and GreenFeed (C-Lock Inc., Rapid City, SD, USA) measurements for 235 cows from 3 breeds, of which only 76 cows from 1 breed were used to calibrate the equation predicting MeY.
- MeP_indirect (in g/d, R^2^ = 0.38), obtained by multiplying the predicted MeI by the observed FPCM associated with the spectra.

Only test-day data between 70 and 200 days in milk were considered, according to Fresco et al. [20]; outside this interval, the validity of the equations predicting MeP_direct, MeI, and MeY was not guaranteed. Due to contractual restrictions, milk MIR spectra were not directly available to us. Instead, predictions were generated by ESTEL (Nancy, France), the IT company in charge of routinely performing all milk MIR spectra predictions for French milk recording companies.

### Adjustment of phenotypes

For each trait, outlier values—defined as those falling outside three standard deviations around the mean—were excluded from the dataset. Univariate repeatability models were applied to estimate environmental effects and then to adjust the phenotypes (i.e. each prediction from the milk MIR spectra), using Genekit software [23]. The pedigree was traced back four generations and contained 903,383 animals. The linear model was the following:

1 $$\mathbf{y}=\mathbf{X}{\varvec{\upbeta}}+\mathbf{Z}\mathbf{a}+\mathbf{W}\mathbf{p}\mathbf{e}+\mathbf{e}$$

where **y** is the vector of CH_4_ predictions; **β** is the vector including the fixed effects of herd x test-day [168,362 classes], year x month of calving [84 classes], age at calving [17 classes], and the covariate days in milk modeled by third-order Legendre polynomials; **X** is the corresponding incidence matrix; $\mathbf{a} \sim N(\mathbf{0},\mathbf{A}{\sigma }_{a}^{2}$) is the vector of breeding values, with **A** the pedigree relationship matrix among individuals and ${\sigma }_{a}^{2}$ the additive genetic variance; $\mathbf{p}\mathbf{e} \sim N(\mathbf{0},\mathbf{I}{\sigma }_{pe}^{2}$) is the vector of permanent environmental effects, with **I** the identity matrix and ${\sigma }_{pe}^{2}$ the permanent environmental variance; **Z** and **W** are the incidence matrices; and $\mathbf{e} \sim N(\mathbf{0},\mathbf{I}{\sigma }_{e}^{2}$) is the vector of residual effects, with ${\sigma }_{e}^{2}$ the residual variance. Variance components were estimated from a subset of 579,753 records from 156,381 cows, already described in Fresco et al. [9]. Genetic covariance between the six CH_4_ traits were estimated using bivariate analyses with Eq. (1). These components were estimated using the AI-REML algorithm, as implemented in Wombat software [24]. The corrected phenotypes were then averaged for each individual for use in the GWAS analyses.

### 50 K genotypes and imputation to whole-genome sequence

Among the cows with phenotypes, 40,609 had genotypes for the 50 K chip and were subsequently used in GWAS analyses. The 50 K genotypes were imputed to whole-genome sequences (WGS) in two steps, similar to the procedure presented by Tribout et al. [25] and Sanchez et al. [26]. Briefly, we first imputed from 50 K to high density (HD) within breed using FImpute software [27], using 776 major French Holstein bulls genotyped with the HD Illumina chip as a reference, and then from HD to WGS with Minimac software [28], using the WGS variants of a multi-breed *Bos taurus* bull population from Run9 of the 1000 Bull Genomes Project [29]. This panel included 3414 bulls from 45 breeds, including 1414 Holsteins. After quality control (MAF > 0.01, Minimac imputation R^2^ > 0.20), 13,366,479 variants were retained for GWAS analyses of the 29 autosomes.

### GWAS analyses

Due to computational challenges arising from the large size of the genomic matrix, we performed the GWAS analyses by randomly splitting the population into two equal sub-populations. Single-trait association analyses were then performed across all 13,307,594 variants (MAF > 0.01) for each of the six predicted CH_4_ traits. All association analyses were performed using the mlma option of GCTA software (version 1.26), which applies a mixed linear model that includes the variant to be tested [30]:

2 $${\mathbf{y}}_{\mathbf{c}}=\mathbf{1}\upmu +\mathbf{x}\text{b}+\mathbf{Z}\mathbf{u}+\mathbf{e}$$

where ${\mathbf{y}}_{{\varvec{c}}}$ is the vector of averaged corrected phenotypes for each of the six CH_4_ traits; $\upmu$ is the overall mean; $\text{b}$ is the additive fixed effect of the variant to be tested for association; $\mathbf{x}$ is the vector of imputed alternate allele dosages, coded from 0 to 2; $\mathbf{u}$ ~ $N$(**0**,$\mathbf{G}{\upsigma }_{\text{u}}^{2}$) is the vector of random polygenic effects, with $\mathbf{G}$ the genomic relationship matrix calculated using the 50 K genotypes and ${\upsigma }_{\text{u}}^{2}$ the polygenic variance, estimated based on the null model (${\mathbf{y}}_{\mathbf{c}}=\mathbf{1}\upmu +\mathbf{Z}\mathbf{u}+\mathbf{e}$) and then fixed while testing for the association between each variant and the trait; and $\mathbf{e}$ ~ $N$(**0**,$\mathbf{I}{\upsigma }_{\text{e}}^{2}$) is the vector of random residual effects, with $\mathbf{I}$ the identity matrix and ${\upsigma }_{\text{e}}^{2}$ the residual variance. **Z** was the incidence matrix.

We combined the two GWAS results by performing a meta-analysis (meta-GWAS) using METAL software [31] with the *STDERR* option, which applies the fixed effects approach. We estimated the inflation factor for each trait (1.10–1.25) and corrected the p-values accordingly. To account for multiple testing, we applied a highly conservative approach, the Bonferroni correction that considered 13,307,594 independent tests in the meta-GWAS. The 5% genome-wide threshold of significance therefore corresponded to a nominal P-value of 3.75 × 10^–9^ (-log10(P) = 8.4) per test. We determined QTL intervals based on a linkage disequilibrium analysis using the correlations between imputed dosages. We applied the iterative procedure described in Sanchez et al. [32]. In short, this procedure selects the variant with the highest -log10(P) per *Bos taurus* autosome (BTA), estimates its linkage disequilibrium (LD) with nearby variants within a user-defined window, and defines the QTL confidence interval based on high-LD variants. A new test statistic evaluates whether other variants' effects are explained by LD with the top variant. Variants meeting this criterion and identified in the QTL confidence interval are removed before the next iteration.

For our study, we used the following parameters in the iterative procedure: a minimal linkage disequilibrium of 0.45, a minimal MAF of 0.01, a minimal imputation R^2^ of 0.2, and a window size set to 4 Mbp. When we observed that the TOP1 variant was in high LD with variants at the edge of the window and that another QTL was detected just outside of the bounds of the window, we performed a second LD analysis using the same iterative procedure with the same parameters on a larger window for the concerned BTAs to determine whether we really had two neighboring QTLs or whether it was a unique QTL with LD at a greater distance. We widened the window to 8 Mbp (BTA1, 3, and 14 for MeP_RC; BTA14 for MeP_direct; BTA14 and 20 for MeI; BTA19 for MeP_FA) or 18 Mbp (BTA14 for MeY and BTA6 for MeP_RC). For each trait, the proportion of genetic variance explained by each QTL (${\sigma }_{u\_QTL}^{2}$) was estimated using ${\sigma }_{u\_QTL}^{2}=100\times (2\times {p}_{ms}\times \left(1-{p}_{ms}\right)\times {\widehat{b}}_{ms}^{2})/{\sigma }_{u}^{2}$, with ${p}_{ms}$ and ${\widehat{b}}_{ms}$ the frequency and the estimated allelic substitution effect, respectively, of the variant with the most significant effect (*ms*) in the QTL.

### Annotation of candidate genes

Genomic regions were defined as regions containing one QTL or multiple QTLs whose confidence intervals overlapped. Within these regions, the variant with the most significant effect, i.e., ranked first in the peak, was referred to as the TOP1. The top ten most significant variants within each peak were referred to as the TOP10. QTLs were annotated using the Ensembl gene set integrating the ARS-UCD1.3 bovine reference genome [33]. Positional candidate genes were defined as those containing at least one variant from the TOP10. To further characterize these variants, the Ensembl Variant Effect Predictor (VEP, [34]) was used to provide detailed functional annotations.

## Results

### Estimation of genetic parameters using a repeatability model

Descriptive statistics for the six CH_4_ traits during the first lactation, measured between 70 and 200 days in milk, are the same as in Fresco et al. [9] and are presented in Table 1. For the four CH_4_ traits measured in g/d, the average emissions varied from 367 to 448 g/d, while mean MeI was 14.0 g/kg of FPCM and mean MeY was 17.8 g/kg of DMI. The genetic, permanent environmental, and residual variances for these traits are detailed in Table 2. Heritability estimates varied from 0.23 to 0.42 depending on the CH_4_ trait (Table 2).

**Table 1:** Descriptive statistics of the methane traits predicted from milk MIR spectra, used for estimation of variance components

Trait	Mean	Standard deviation	Minimum	Maximum
MeP_direct (g/d)	420	41.2	227	572
MeI (g/kg of FPCM)	14.0	1.82	7.4	19.7
MeY (g/kg of DMI)	17.8	1.40	13.4	22.9
MeP_indirect (g/d)	367	80.2	95	630
MeP_FA (g/d)	448	43.61	295	570
MeP_RC (g/d)	427	41.76	292	577

**Table 2:** Variance and heritability estimates for the six methane traits predicted from milk MIR spectra

Trait	Genetic variance	Permanent environment variance	Residual variance	Heritability
MeP_direct (g/d)	291	139	310	0.39 (0.006)
MeI (g/kg of FPCM)	0.36	0.14	0.56	0.34 (0.006)
MeY (g/kg of DMI)	0.24	0.68	0.31	0.35 (0.006)
MeP_indirect (g/d)	690	1204	1050	0.23 (0.007)
MeP_FA (g/d)	274	160	459	0.31 (0.006)
MeP_RC (g/d)	364	133	368	0.42 (0.006)

### Genome-wide association studies for the six predicted CH_4_ traits

**Fig. 1:**
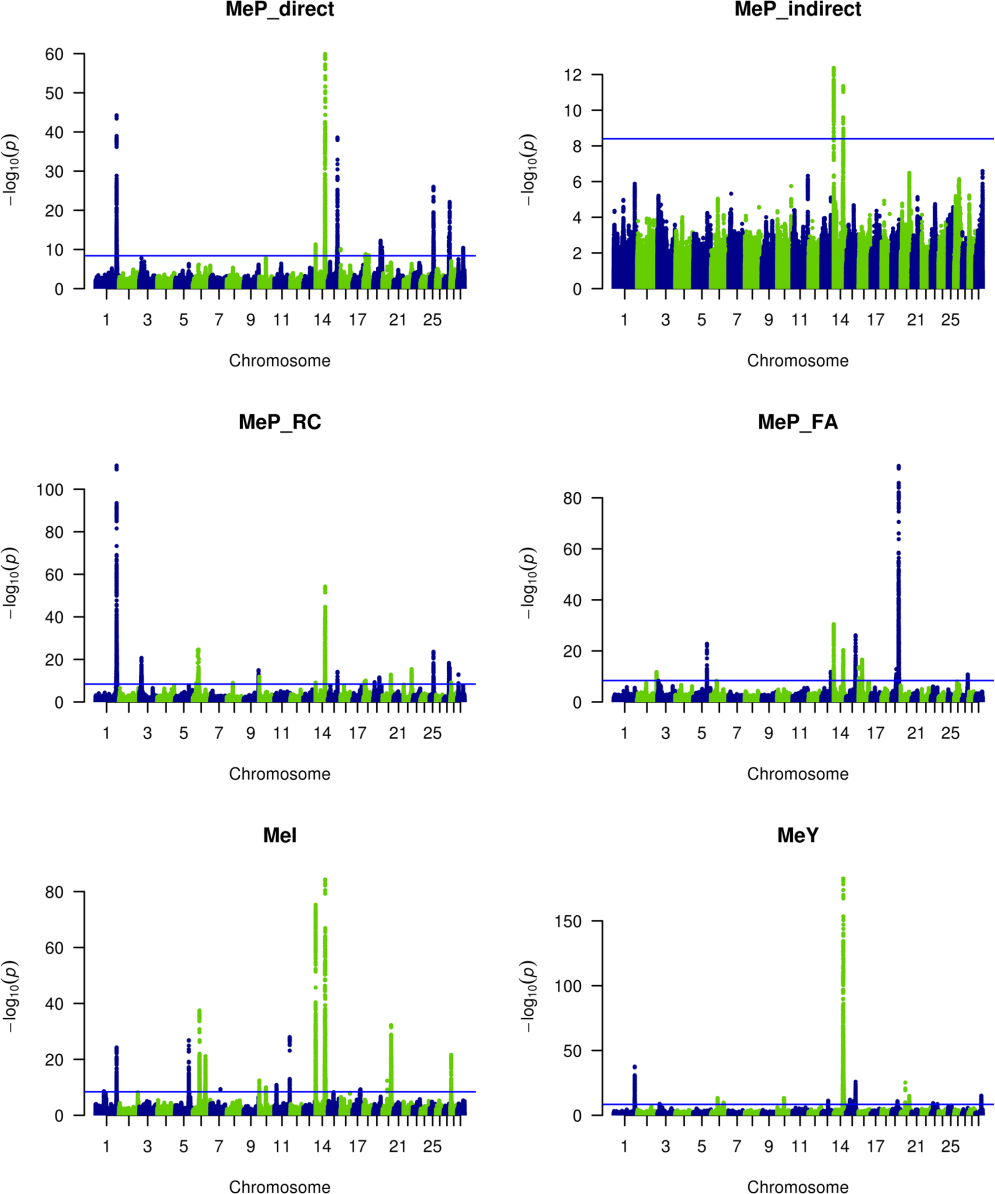
-log10(P) values plotted against the position of variants on *Bos taurus* autosomes for the meta-analyses of methane traits predicted from milk mid-infrared spectra. MeP_direct = prediction of methane production in g/d [20], MeI = prediction of methane intensity in g/kg of fat- and protein-corrected milk [20], MeY = prediction of methane yield in g/kg of dry matter intake [20], MeP_indirect = predicted MeI multiplied by the observed fat- and protein-corrected milk [20], MeP_FA = prediction of methane production in g/d [18], and MeP_RC = prediction of methane production in g/d [19]

The results of the meta-GWAS combining the single-trait GWAS analyses of the two randomly divided groups of individuals are presented in Fig. 1. The number of variants with a genome-wide significant effect (− log10(P) ≥ 8.4) varied from 157 to 9758 depending on the CH_4_ trait in question (Table 3). There were between 1 and 3422 significant variants per QTL, with an average of 313 variants (standard deviation = 618). Notably, 86.5% (90 of 104) and 92.0% (897 of 975) of the TOP1 and TOP10 variants were imputed, respectively. Only 4.9% of the TOP1 and 5.1% of the TOP10 variants presented a MAF between 0.01 and 0.02. The maximum -log10(P) value observed was 181, for a variant on BTA14 associated with MeY.

**Table 3:** Number of significant variants, number of QTLs, and percentage of genetic variance explained

Trait	Number of significant variants	Number of QTLs	SE of QTL effect	% of genetic variance explained by the QTL		
				Minimum	Maximum	Total
MeP_direct (g/d)	4649	17	0.01–0.74	0.12	4.0	12.0
MeI (g/kg of FPCM)	9758	25	0.01–0.07	0.16	7.1	24.7
MeY (g/kg of DMI)	7922	17	0.01–0.02	0.14	12.5	20.7
MeP_indirect (g/d)	157	2	0.45–0.57	0.63	0.9	1.5
MeP_FA (g/d)	2911	15	0.15–0.42	0.12	3.1	11.7
MeP_RC (g/d)	7186	28	0.15–0.90	0.14	6.4	23.0

We detected 2 to 28 QTLs per CH_4_ trait, for a total of 104 QTLs across all six CH_4_ traits (Table 4a, b, c, d, and e). These QTLs were distributed across 24 of the 29 autosomes, with 1 to 15 QTLs per BTA. The confidence intervals of the QTLs varied from 2 to 11,980,490 bp, and 3 QTL were defined by a single variant. When neighboring QTLs had overlapping confidence intervals, we combined them into genomic regions; this resulted in the identification of 57 distinct genomic regions, each containing between 1 and 8 QTLs. Of these, 22 genomic regions contained at least 2 QTLs affecting one or multiple CH_4_ traits. Four regions were of particular interest: one located on BTA14 at ~ 64 Mbp (region 28), which was associated with all six CH_4_ traits; one located on BTA14 at ~ 0.5 Mbp (region 26), which was associated with five of the six CH_4_ traits; and two regions located on BTA1 at ~ 142 Mbp (region 2) and BTA15 at ~ 65 Mbp (region 32), which were associated with four of the six CH_4_ traits.

**Table 4:** QTLs identified for predicted methane traits

Region	Trait	BTA	QTL	Confidence interval of the QTL				Variant with the most significant effect (all had an imputation R^2^ of 1)					Name of genes TOP10
				Start (bp)	End (bp)	N variants	N genes	Position (bp)	-log_10_P	b	SE	Name of gene TOP1	
1	MeI	1	1	57,130,469	57,632,402	9	4	57,631,653	8.5	0.06	0.01	*ENSBTAG00000057099*	*CD200, ENSBTAG00000060686, ENSBTAG00000057099, ENSBTAG00000001235*
2	MeP_RC	1	5	139,923,727	146,447,185	2355	47	142,814,697	111.2	− 5.80	0.22	*SLC37A1*	*SLC37A1*
2	MeP_direct	1	4	141,872,915	144,306,817	1183	13	142,814,697	44.1	− 3.04	0.18	*SLC37A1*	*SLC37A1*
2	MeY	1	3	141,915,045	144,306,817	1177	12	142,806,542	37.5	− 0.08	0.01	*SLC37A1*	*SLC37A1*
2	MeI	1	2	142,591,987	142,595,307	7	0	142,594,565	13.2	0.06	0.01		
2	MeI	1	6	142,604,730	142,991,203	615	6	142,879,906	24.0	0.09	0.01		*SLC37A1*
3	MeP_FA	2	7	131,103,444	131,359,941	354	7	131,245,133	11.7	− 2.44	0.30		*ENSBTAG00000068711*
4	MeP_RC	3	10	13,177,910	17,220,237	65	5	17,171,600	12.2	− 4.91	0.59		*ENSBTAG00000065185*
*4*	*MeP_RC*	*3*	*8*	*13,672,338*	*17,166,665*	*149*	*28*	*15,470,670*	*20.8*	*3.28*	*0.30*	*EFNA1*	*SLC50A1, EFNA1, ENSBTAG00000061595*
4	MeY	3	9	15,504,899	15,602,932	40	7	15,555,371	8.8	0.06	0.01	*DCST1*	*ADAM15, DCST1, DCST2, ZBTB7B*
5	MeI	5	11	92,843,362	94,221,469	16	1	93,375,037	10.0	0.09	0.01		*EPS8*
5	MeI	5	12	93,391,179	93,749,199	205	2	93,516,066	26.6	− 0.10	0.01	*MGST1*	*MGST1*
*5*	*MeP_FA*	*5*	*13*	*93,512,771*	*93,751,239*	*124*	*2*	*93,516,319*	*22.7*	− *2.42*	*0.21*	*MGST1*	*MGST1*
6	MeP_RC	6	14	23,596,912	23,604,273	3	1	23,597,653	8.9	5.49	0.78	*PPP3CA*	*PP3CA*
7	MeP_RC	6	15	33,528,107	35,173,221	31	3	34,288,754	24.1	− 9.36	0.78	*CCSER1*	*CCSER1, ENSBTAG00000065318, ENSBTAG00000065966*
8	MeP_RC	6	16	35,571,947	36,587,776	5	3	35,594,567	11.7	− 4.82	0.59	*ENSBTAG00000062289*	*GPRIN3, ENSBTAG00000062289, ABCG2*
9	MeP_RC	6	17	36,955,669	37,003,208	17	2	36,997,477	11.6	4.40	0.54		*ENSBTAG00000065096*
10	MeP_RC	6	18	38,148,560	38,485,730	34	0	38,454,876	16.5	− 4.28	0.44		
11	MeP_RC	6	19	38,485,998	40,748,806	7	1	38,494,188	24.8	10.00	0.83		*KCNIP4*
12	MeI	6	20	44,250,735	44,262,727	3	0	44,262,048	8.6	− 0.05	0.01		
13	MeI	6	21	44,283,395	44,308,333	8	0	44,299,242	14.7	− 0.06	0.07		
14	MeI	6	22	44,329,162	46,642,860	856	19	44,747,933	37.1	0.11	0.01		*ENSBTAG00000064660*
14	MeY	6	23	44,674,698	45,967,046	48	3	45,060,087	13.3	0.06	0.01	*ENSBTAG00000064660*	*ENSBTAG00000064660*
15	MeI	6	24	82,582,052	82,605,868	18	0	82,593,766	8.5	0.05	0.01		
16	MeI	6	25	83,640,723	85,702,894	369	17	85,503,742	20.0	− 0.10	0.01	*ENSBTAG00000060764*	*ENSBTAG00000060764, CSN1S2*
16	MeY	6	28	85,424,759	86,448,746	68	7	86,221,365	9.6	0.07	0.01	*GRSF1*	*CSN1S1, GRSF1*
16	MeI	6	26	85,703,928	85,703,928	1	0	85,703,928	9.0	− 0.06	0.01		
16	MeI	6	27	85,736,321	86,158,733	35	4	85,736,621	10.8	− 0.10	0.01	*CABS1*	*CABS1*
17	MeI	7	29	68,579,274	68,592,765	58	2	68,579,369	9.3	0.09	0.01	*ENSBTAG00000057983*	*ENSBTAG00000057983, ENSBTAG00000059254*
18	MeP_RC	8	30	41,366,883	42,068,650	11	0	41,366,883	9.1	1.94	0.27		
19	MeP_RC	9	31	100,858,075	102,458,517	450	13	101,345,396	15.0	− 2.11	0.23	*ENSBTAG00000059544*	*ENSBTAG00000059544*
20	MeI	10	32	1,341,660	2,214,558	119	2	2,187,345	12.3	0.04	0.01		*APC, ENSBTAG00000058588*
20	MeP_RC	10	33	2,137,692	2,214,558	116	0	2,191,110	11.9	1.22	0.15		
21	MeY	10	34	46,048,106	46,548,638	438	7	46,051,704	13.2	− 0.08	0.01	*DAPK2*	*DAPK2, ENSBTAG00000060901, USP3*
*21*	*MeI*	*10*	*35*	*46,088,320*	*46,548,638*	*172*	*5*	*46,541,616*	*9.9*	*− 0.06*	*0.01*	*ENSBTAG00000057172*	*USP3, ENSBTAG00000057172*
*22*	*MeI*	*11*	*36*	*14,229,710*	*14,642,556*	*128*	*2*	*14,229,992*	*10.6*	*− 0.05*	*0.01*		*MEMO1*
23	MeI	11	37	103,226,637	103,286,957	374	4	103,254,495	27.7	− 0.12	0.01		*ENSBTAG00000014678*
24	MeY	13	38	45,882,199	47,070,574	194	6	46,366,318	11.3	0.05	0.01		*GTPBP4*
25	MeP_FA	13	39	64,053,550	64,262,227	52	3	64,147,879	11.7	− 1.86	0.23	*NCOA6*	*NCOA6, ACSS2, GSS*
26	MeI	14	46	180,920	3,104,982	220	21	1,704,582	26.7	− 0.09	0.01	*PSCA*	*PSCA, ENSBTAG00000026340, ABDRB1, TSNARE1*
*26*	*MeI*	*14*	*44*	*233,681*	*993,201*	*522*	*37*	*608,230*	*73.7*	*− 0.26*	*0.01*	*DGAT1*	*CPSF1, ENSBTAG00000000857, SCRT1, DGAT1*
*26*	*MeP_FA*	*14*	*41*	*264,797*	*993,201*	*324*	*33*	*550,784*	*30.3*	*− 4.57*	*0.35*	*CPSF1*	*CPSF1, ADCK5, DGAT1*
*26*	*MeP_direct*	*14*	*43*	*384,242*	*901,826*	*111*	*17*	*568,472*	*11.2*	*3.05*	*0.37*	*ADCK5*	*ADCK5, DGAT1, HSF1*
26	MeP_indirect	14	42	384,242	707,908	128	16	565,009	12.5	− 4.11	0.45	*ADCK5*	*ADCK5, DGAT1, HSF1*
26	MeP_RC	14	40	497,507	955,774	73	11	500,872	9.2	− 3.24	0.45	*TONSL*	*TONSL, CPSF1, ENSBTAG00000000857, SCRT1, DGAT1, HSF1*
27	MeP_FA	14	45	995,464	1,915,755	136	12	1,028,657	13.5	− 1.70	0.20	*IQANK1*	*IQANK1, FAM83H, ZC3H3, ADGRB1*
28	MeY	14	50	59,747,181	71,727,670	3422	59	64,752,983	181.3	0.42	0.01	*VPS13B*	*VPS13B, ENSBTAG00000055757*
28	MeI	14	47	61,219,666	68,426,997	2910	48	64,454,721	83.2	0.23	0.01	*VPS13B*	*VPS13B, ENSBTAG00000055757*
28	MeP_direct	14	51	61,457,257	65,916,772	1760	31	64,779,893	59.7	− 8.29	0.42	*VPS13B*	*VPS13B, ENSBTAG00000055757*
28	MeP_RC	14	52	61,527,782	65,872,284	1411	31	64,926,343	54.5	− 13.86	0.76	*VPS13B*	*VPS13B, ENSBTAG00000055757*
28	MeP_FA	14	49	63,173,429	65,641,447	320	22	64,672,856	20.1	− 4.45	0.42	*VPS13B*	*VPS13B, ENSBTAG00000055757*
28	MeP_indirect	14	48	64,043,186	65,402,152	29	5	64,672,856	11.3	4.96	0.57	*VPS13B*	*VPS13B, ENSBTAG00000055757*
29	MeY	15	53	27,315,640	28,691,118	212	17	27,646,671	11.4	0.05	0.01	*SIK3*	*ENSBTAG00000067623, SIK3*
30	MeY	15	54	53,137,808	53,767,920	373	8	53,359,144	11.1	0.07	0.01	*PAAF1*	*MRPL48, COA4, PAAF1, DNAJB13*
31	MeP_direct	15	55	63,019,643	63,055,774	71	1	63,055,774	9.9	1.02	0.13	*ENSBTAG00000052796*	*ENSBTAG00000052796*
32	MeP_direct	15	61	63,736,668	65,705,004	252	10	65,333,701	38.5	− 2.46	0.16		*EHF*
32	MeP_FA	15	58	63,804,912	65,346,043	200	5	65,312,559	26.0	1.89	0.15		*EHF*
32	MeY	15	56	64,794,022	66,090,611	67	3	64,794,049	11.9	0.04	0.01	*ABTB2*	*ABTB2*
*32*	*MeY*	*15*	*59*	*64,918,334*	*65,678,067*	*251*	*9*	*65,323,234*	*25.9*	− *0.05*	*0.01*		*ENSBTAG00000059416, ENSBTAG00000063234, EHF*
32	MeP_RC	15	57	65,227,767	65,239,009	3	0	65,239,009	8.6	1.23	0.18		
32	MeP_RC	15	60	65,242,698	65,338,417	32	0	65,324,226	14.3	1.68	0.19		
32	MeP_FA	15	62	65,355,672	65,515,606	13	3	65,355,672	9.1	− 2.01	0.29		*PDHX*
32	MeP_direct	15	63	66,074,954	66,096,784	12	0	66,089,302	10.6	− 1.17	0.15		
33	MeP_FA	16	65	1,756,944	1,907,186	19	1	1,786,309	13.3	− 2.87	0.33		*SOX13*
33	MeP_direct	16	64	1,784,784	1,791,556	8	0	1,786,309	10.1	2.56	0.33		
34	MeP_FA	16	66	24,251,952	25,510,748	148	2	24,280,507	16.5	− 1.60	0.17	*MTARC1*	*MTARC1*
35	MeI	17	67	51,586,460	51,586,460	1	0	51,586,460	8.5	0.04	0.01		
36	MeI	17	68	53,714,812	54,541,221	51	3	53,794,372	9.2	0.08	0.01		*KDM2B, RNF34*
37	MeP_RC	18	69	8,826,358	10,690,398	10	2	8,828,778	9.6	6.42	0.88	*ENSBTAG00000069108*	*ENSBTAG00000069108, ENSBTAG00000057902*
38	MeP_RC	18	71	15,689,862	16,420,403	8	2	16,155,339	10.1	6.80	0.90		*ITFG1, PHKB*
38	MeP_direct	18	70	16,152,862	16,155,339	2	0	16,155,339	8.8	5.32	0.74		
39	MeP_RC	19	72	10,283,388	10,294,221	5	0	10,294,221	9.3	1.17	0.16		
40	MeP_FA	19	75	32,335,650	34,947,083	4	2	34,947,083	8.6	1.57	0.23	*PLD6*	*COPS3, PLD6*
40	MeP_FA	19	73	33,521,281	33,536,010	30	1	33,527,729	8.7	− 2.25	0.33	*PECC1*	*PECC1*
40	MeP_FA	19	74	33,666,818	34,856,338	28	12	34,729,508	12.7	2.17	0.26	*RAI1*	*ENSBTAG00000063426, EPN2, ENSBTAG00000066713, RAI1*
41	MeY	19	76	42,238,476	42,626,158	688	14	42,265,893	10.9	0.07	0.01	*RAB5C*	*RAB5C, STAT3*
41	MeP_RC	19	77	42,238,476	42,626,158	689	14	42,281,725	11.6	− 2.81	0.35		*RAB5C, ENSBTAG00000065500*
42	MeP_FA	19	80	47,997,400	54,407,498	1102	58	50,775,172	91.5	− 4.29	0.18		*CCDC57, FASN*
42	MeP_direct	19	78	49,996,881	50,198,903	17	2	50,198,788	10.0	1.73	0.23		*UTS2R*
42	MeP_direct	19	79	50,207,005	50,780,248	97	4	50,721,397	12.2	− 1.66	0.19	*CCDC57*	*CCDC57, FASN*
43	MeP_direct	19	81	60,404,660	61,078,226	264	2	60,441,152	9.7	− 1.26	0.17		*ENSBTAG00000059677, ENSBTAG00000063788*
*44*	*MeY*	*20*	*82*	*31,462,828*	*32,320,669*	*13*	*3*	*31,888,449*	*25.2*	*0.13*	*0.01*	*GHR*	*GHR, ENSBTAG00000067324, ENSBTAG00000059721*
*44*	*MeI*	*20*	*83*	*32,018,037*	*32,103,408*	*3*	*2*	*32,018,037*	*9.1*	*− 0.09*	*0.01*	*GHR*	*GHR, ENSBTAG00000067324*
45	MeI	20	85	55,290,311	60,131,394	2933	16	58,195,251	31.9	0.12	0.01		*ANKH*
45	MeP_RC	20	84	55,514,835	57,379,190	770	10	56,489,500	13.0	2.86	0.33	*MYO10*	*MYO10, RETREG1*
*45*	*MeY*	*20*	*86*	*58,191,503*	*59,878,202*	*830*	*3*	*58,377,254*	*14.6*	*0.06*	*0.01*	*ANKH*	*ANKH, DNAH5*
46	MeP_RC	22	87	53,951,400	54,565,136	138	3	54,475,210	14.2	− 3.07	0.34	*ATP2B2*	*ATP2B2*
47	MeP_RC	22	88	54,611,746	54,677,719	42	1	54,629,523	15.5	1.44	0.15	*ATP2B2*	*ATP2B2*
48	MeY	23	89	16,740,006	17,219,907	2	1	16,740,006	9.4	0.05	0.01	*PTK7*	*PTK7*
49	MeY	23	90	45,223,069	45,229,851	6	1	45,223,636	8.6	0.04	0.01	*ELOVL2*	*ELOCL2*
50	MeP_direct	25	91	25,899,872	25,899,872	1	0	25,899,872	8.9	1.18	0.16		
51	MeP_direct	25	93	25,904,431	27,723,625	621	50	26,061,709	26.0	− 2.29	0.18	*IL27*	*ENSBTAG00000064180, ENSBTAG00000050361, IL27*
51	MeP_RC	25	92	25,906,818	27,473,500	495	45	26,048,468	23.7	− 2.60	0.22	*CLN3*	*CLN3*
*52*	*MeP_RC*	*27*	*95*	*36,353,153*	*36,617,333*	*109*	*10*	*36,522,458*	*18.4*	*2.42*	*0.23*		*GPAT4*
*52*	*MeP_direct*	*27*	*94*	*36,378,091*	*36,558,128*	*32*	*4*	*36,522,458*	*13.2*	*1.69*	*0.19*		*GINS4, ENSBTAG00000058864, GPAT4, NKX6-3*
53	MeP_direct	27	96	40,911,676	41,646,056	158	2	41,391,206	22.1	− 1.92	0.16	*ENSBTAG00000056465*	*ENSBTAG00000056465*
53	MeP_RC	27	97	41,375,472	41,627,439	141	2	41,391,206	16.1	− 1.94	0.20	*ENSBTAG00000056465*	*ENSBTAG00000056465*
53	MeP_FA	27	98	41,379,852	41,601,820	57	2	41,392,181	10.7	1.25	0.16	*ENSBTAG00000056465*	*ENSBTAG00000056465*
54	MeP_direct	27	99	41,737,999	41,971,860	5	3	41,971,860	8.9	− 1.68	0.23	*ENSBTAG00000064242*	*THRB, ENSBTAG00000065779, ENSBTAG00000064242*
*55*	*MeI*	*28*	*101*	*6,192,309*	*7,165,451*	*125*	*2*	*6,458,572*	*21.4*	*0.06*	*0.01*	*KCNK1*	*KCNK1*
55	MeP_RC	28	100	6,457,299	6,529,103	8	1	6,457,425	9.2	3.33	0.46	*KCNK1*	*KCNK1*
56	MeP_RC	29	102	9,486,040	9,545,883	9	1	9,502,594	13.0	1.76	0.20		*PICALM*
57	MeY	29	104	40,666,856	42,245,387	93	12	41,325,720	15.2	0.17	0.02		*SLC22A8, ENSBTAG00000037570*
57	MeP_direct	29	103	41,149,123	42,009,447	55	7	41,319,432	10.4	− 3.83	0.49	*SLC22A8*	*SLC22A6, SLC22A8*

The genotyped TOP1 variants are indicated in italics

The CH_4_ traits with the highest number of genomic regions in common were MeP_direct and MeP_RC (8 genomic regions), followed by MeP_RC and MeY (7), MeI and MeY (7), MeP_direct and MeP_FA (6), and MeP_RC and MeI (6) (Table 5).

**Table 5:** Genetic correlations and number of genomic regions shared between pairs of predicted methane traits

Trait	MeP_direct	MeI	MeY	MeP_indirect	MeP_FA	MeP_RC
MeP_direct (g/j)		0.25	0.81	0.36	0.21	0.62
MeI (g/kg of FPCM)	3		0.38	0.52	− 0.15	0.18
MeY (g/kg of DMI)	4	7		0.36	0.13	0.50
MeP_indirect (g/d)	2	2	1		0.03	0.20
MeP_FA (g/d)	6	3	2	2		0.24
MeP_RC (g/d)	8	6	7	2	4	

The percentage of genetic variance explained for each trait varied from 1.5% to 24.7% (Table 3). The variants explaining the highest percentage of genetic variance were found in QTL 51 (4.0%) for MeP_direct; QTL 80 (3.1%) for MeP_FA; QTL 52 (6.4%) for MeP_RC; QTL 42 (0.9%) for MeP_indirect; QTL 44 (7.1%) for MeI; and QTL 50 (12.5%) for MeY. Overall, 30 QTLs contained a TOP1 variant with a -log10(P) value exceeding 20 that we defined as highly significant (Table 4). These included 6 highly significant QTLs for MeP_RC, 5 for MeP_FA, 5 for MeP_direct, none for MeP_indirect, 10 for MeI, and 4 for MeY. Several of these highly significant QTLs were located in the same genomic regions: region 2 on BTA1 at ~ 142 Mbp for MeP_RC, MeP_direct, MeI, and MeY; region 26 on BTA14 at ~ 0.5 Mbp for MeP_FA and MeI; region 32 on BTA15 at ~ 65 Mbp for MeP_direct, MeP_FA, and MeY; and region 51 on BTA25 at ~ 26 Mbp for MeP_RC and MeP_direct.

### Functional annotation of GWAS peaks

The total number of genes within the confidence intervals of at least one QTL was 937, with an average of 9 genes per QTL and a range from 0 to 59; in 16 QTLs, no genes were highlighted. Positional candidate genes were defined as those containing at least one of the TOP10 variants for each QTL. We identified 35, 35, 26, 28, 37, and 5 positional candidate genes for MeI, MeY, MeP_direct, MeP_FA, MeP_RC, and MeP_indirect, respectively.

The genomic region associated with all six CH_4_ traits contained the *VPS13B* gene, while the genomic regions associated with four or five of the CH_4_ traits contained the *DGAT1*, *SLC37A1*, and *EHF*/*PDHX* genes. The QTLs explaining the highest percentage of genetic variance were located in the genomic regions containing the *VPS13B*, *DGAT1*, and *FASN* and *CCDC57* genes.

The TOP10 variants for each QTL were annotated using VEP, and some had multiple annotations (Table 6). Between 0% (MeP_indirect) and 18.4% (MeI) of the TOP10 variants were located in intergenic regions, while 47.3% (MeP_direct) to 72.1% (MeP_indirect) were located in intronic regions. Among the TOP10 variants located in coding regions, 1.2% to 2.5% were synonymous variants, and between 0.6% and 5.5% corresponded to missense mutations. Among these missense mutations, some were classified as deleterious based on their SIFT score: two transcripts of the same variant of the *VPS13B* gene for MeI, MeY, MeP_RC, and MeP_direct; two transcripts of two variants of the gene *ENSBTAG00000050361* for MeP_direct; and one transcript of a variant of the *PSCA* gene for MeI. Additionally, we identified a frameshift variant in the *PLD6* gene within QTL 75 on BTA19 at ~ 34 Mbp for MeP_FA.

**Table 6:** Functional annotation of the 10 most significant variants of each QTL

Trait	MeP_direct (g/d)		MeI (g/kg of FPCM)		MeY (g/kg of DMI)		MeP_indirect (g/d)		MeP_FA (g/d)		MeP_RC (g/d)	
	N	%	N	%	N	%	N	%	N	%	N	%
Variants	134		194		142		18		134		226	
Annotations	383		441		394		68		422		626	
Intergenic	45	11.7	81	18.4	21	5.3			27	6.4	81	13.0
Intron	181	47.3	233	52.8	282	71.6	62	72.1	240	56.9	414	66.2
Downstream_gene	78	20.4	53	12.0	24	6.1	16	18.6	58	13.8	46	7.4
Upstream_gene	42	11.0	30	6.8	27	6.8	6	6.9	57	13.5	39	6.2
5_prime_UTR			11	2.5	14	3.5					11	1.7
3_prime_UTR	3	0.8	6	1.4	3	0.8			14	3.3	7	1.1
Regulatory region	4	1.0	5	1.1	7	1.8			7	1.7	9	1.4
Non_coding_transcript_exon	1	0.2							1	0.2	3	0.5
Synonymous	8	2.1	11	2.5	9	2.3	1	1.2	8	1.9	12	1.9
Missense	21	5.5	11	2.5	5	1.3			9	2.1	4	0.6
Frameshift									1	0.2		

## Discussion

To the best of our knowledge, this study is the first GWAS of imputed whole-genome sequences to use such a large population of dairy cattle to investigate CH_4_ emissions predicted from routinely collected milk MIR spectra.

### Genomic regions shared between CH_4_ traits

We found many genomic regions in common among the six CH_4_ traits. Out of the 57 genomic regions identified in our study, 21 were shared by at least two CH_4_ traits, corresponding to 67 QTLs of the 104 identified. The CH_4_ traits that shared the highest number of regions (8) were MeP_RC and MeP_direct, which is consistent with their methodological similarity: they both represent CH_4_ emissions in g/d and their prediction equations were calibrated using the same methodology [19, 20]. The number of shared genomic regions was not related to the genetic correlations between the CH_4_ traits. Indeed, MeP_RC and MeP_direct [genetic correlation of 0.62 (Table 5)] shared 8 genomic regions, while MeP_direct and MeY [genetic correlation of 0.81 (Table 5)] shared 4, and MeP_FA and MeP_direct [genetic correlation of 0.21 (Table 5)] shared 6. Interestingly, the results obtained for MeP_indirect were notably different from those of the other CH_4_ traits. In this study, MeP_indirect was associated with only two QTLs; these included the genes *VPS13B* and *DGAT1* which were linked with multiple CH_4_ traits in this study.

In contrast to our results, which revealed multiple genomic regions in common among the CH_4_ traits investigated, most previously published GWAS analyses of multiple CH_4_ traits (expressed in different units or predicted using different equations) have reported only a few shared genomic regions; such studies have examined MeP and MeY predicted from milk MIR spectra [35] as well as direct measurements of MeP, MeI, and MeY [10, 11].

### Genomic regions shared with different previously published GWAS studies of CH_4_ traits

The genetic architecture of CH_4_ emissions has previously been studied using direct CH_4_ measurements [10–13, 35] or CH_4_ predicted from traits related to feed intake [15–17], the concentration of volatile fatty acids in the rumen [14], milk fat composition [35], and milk MIR spectra [35] in both dairy and beef breeds. Given the diversity of these approaches and the scarcity of shared regions among previous studies, we anticipated that there would be little overlap between the genomic regions highlighted in this study and those reported in the literature. Indeed, only three of the genomic regions identified here were among those described in the literature. Genomic region 42 on BTA19 at ~ 50 Mbp, which in the present study was associated with MeP_direct and MeP_FA and for which *FASN* and *CCDC57* were the most probable candidate genes, was also highlighted in the studies of van Engelen [35] and Manzanilla-Pech et al. [11], which examined CH_4_ emissions in g/kg of DMI and g/d, respectively. Genomic region 26 on BTA14 at ~ 0.5 Mbp, which contained the *DGAT1* gene, was associated with all predicted CH_4_ traits except MeY in our study; similarly, this region was reported to be associated with CH_4_ emissions in both g/d and g/kg of DMI by van Engelen [35]. Finally, genomic region 28 on BTA14 at ~ 64 Mbp was associated with all six predicted CH_4_ traits analyzed here, with *VPS13B* being the most likely candidate gene; this region was also identified in the study of Calderón-Chagoya et al. [13], which used CH_4_ emissions expressed in mg/L.

The small number of genomic regions shared among studies might be the result of the different methods used for measuring CH_4_ emissions, which are known to have correlations of less than one [36, 37]. Another explanation could be that previous GWAS analyses were based on smaller sample sizes (ranging from 150 to 1962 animals), which limited their detection power. Most of these studies used 50 K genotypes, whereas sequence data, like those used in our study, increase the resolution of QTL detection [38]. Additionally, differences in breed composition among studies (Holstein cows [10–12, 14, 15], beef breeds [10, 16, 17], combination of purebred, crossbred, and Zebu cattle [13]) likely affect the results as well, as different QTLs may segregate in different breeds. This hypothesis is supported by the results of Manzanilla-Pech et al. [10], who highlighted that different genes are involved in the expression of CH_4_ traits in Angus and Holstein breeds.

### CH_4_ traits are associated with genes related to milk production and composition

Among the positional candidate genes identified in our study, many have been previously confirmed to be associated with milk traits [25, 39], traits predicted from milk MIR spectra [26, 40], and specific wavelengths of milk MIR spectra [41]. Among these, we notably found *VPS13B*, *SLC37A1*, *DGAT1*, *FASN, CCDC57*, *ANKH, PICALM, MTARC1, GPAT4, DNAJB13, KCNK1, MGST1, USP3, GHR*, and *THRB* genes. These genes were frequently present in the genomic regions containing the most significant variants (-log10(P) ≥ 20), the region that explained the highest percentage of genetic variance for the six CH_4_ traits, or those that were shared by multiple CH_4_ traits. For instance, genomic region 28 on BTA14 at ~ 64 Mbp, which was shared by all CH_4_ traits, included the candidate gene *VPS13B*; genomic region 26 on BTA14 at ~ 0.5 Mbp, which was associated with five CH_4_ traits, included the candidate gene *DGAT1*; and genomic region 2 on BTA1 at ~ 142 Mbp, which was associated with four CH_4_ traits, included the candidate gene *SLC37A1*. Additionally, TOP10 variants from the *VPS13B* gene were missense mutations classified as deleterious by the SIFT score for the traits MeI, MeY, MeP_RC, and MeP_direct.

Independent studies using CH_4_ emissions collected with Sniffers corroborate two of the genes identified in our genomic regions. Manzanilla-Pech et al. [11] identified a SNP significantly associated with MeP within our genomic region 42 (BTA 19 ~ 50 Mbp) for which *FASN* is the most probable candidate gene, and both Pszczola et al. [12] and Calderón-Chagoya et al. [13] identified SNPs associated with MeP less than 1 Mbp before the region. Calderón-Chagoya et al. [13] also identified a SNP within the genomic region 28 (BTA 14 ~ 64 Mbp) for which the candidate gene is *VPS13B*. Additionally, using CH_4_ emissions predicted from milk FA or milk MIR spectra, van Engelen [35] found MeP and MeY to be associated with the *DGAT1* gene.

Of these genes, *FASN* and *DGAT1* could be potential functional candidate genes because they are involved in biological relationships underlying the prediction of CH_4_ from milk MIR spectra. The volatile fatty acids (VFA) produced by the rumen bacteria during fermentation are correlated with hydrogen production, and thus CH_4_ production. VFAs are absorbed by the ruminal epithelium and transferred to the mammary gland, where acetate and butyrate are used as substrates for the de novo synthesis of short-chain fatty acids (SCFA) in milk [42, 43]. The *FASN* gene encodes the fatty acid synthase protein, which is involved in the production of SCFAs in the mammary gland from acetyl-CoA and butyryl-CoA derived from acetate and butyrate [44]. The *DGAT1* gene encodes the diacylglycerol O-acyltransferase 1 protein which produces triacylglycerol from glycerol and SCFAs [44, 45]. Additionally, here we identified an interesting QTL on BTA13, associated with MeP_FA, in which the candidate gene was *ACSS2*, whose protein is involved in the production of acetyl-CoA from acetate in the mammary gland [44].

### Candidate genes for CH_4_ emissions related to rumen microbiota

The rumen microbiota is known to differ between low and high CH_4_-emitters [46, 47] and to be influenced by host genetics [46, 48, 49]. Although one study found that the effect of host genetics on CH_4_ emissions was almost independent of the effect of host genetics on the microbiota [50], others have proposed developing genetic selection on rumen microbiota to reduce CH_4_ emissions [51–53]. Multiple studies have been conducted on the relationships between a host’s genes and its microbiota, including GWASs on beef cattle [48, 54], dairy cattle [46, 55], and sheep [56], but there has been little overlap in the QTLs identified in different studies. We looked for overlapping genomic regions between our GWAS analyses of CH_4_ predicted from milk MIR spectra and the GWAS analyses in the literature to identify possible candidate genes based on biological interpretation. In particular, some of the loci highlighted by Wang et al. [56] are located in the genomic regions identified in our study, which include the candidate genes *EPS8*, *PPP3CA*, *CCSER1*, *KCNIP4*, *NCOA6*, *ABTB2*, *ITFG1*, *PTK7*, and *KCNK1*.

*KCNIP4* may be of particular interest as it was a candidate gene in a highly significant QTL for MeP_RC. Moreover, the corresponding genomic region was also found in another study to be associated with members of the bacterial genera *Prevotella*, *Ruminococcus*, and *Eubacterium* [56]. These bacteria can influence VFA and hydrogen production in the rumen, in the case of *Prevotella* by producing propionate––an alternative to hydrogen production [57]––when digesting starch [58, 59], or, in the case of *Ruminococcus* and *Eubacterium*, by producing acetate and butyrate—and thus hydrogen—when digesting cellulose [58, 59]. Through their influence on VFA and hydrogen production, these bacteria may be able to influence CH_4_ production. Interestingly, they have been found to be overrepresented in the rumen of either high-emitting animals (*Ruminococcus* and *Eubacterium* [51, 60, 61]) or low-emitting animals (*Prevotella* [47, 60, 61]). Other candidate genes in our study were also identified in regions associated with these bacterial genera, such as the gene *CCSER1* (*Prevotella*) and the genes *EPS8*, *PPP3CA*, *NCOA6*, and *ABTB2* (*Eubacterium*) [56].

### Quality of the data

In this study, we conducted GWAS using WGS data from a population of over 40,000 cows, which provided high-resolution detection power for detecting QTLs. It is particularly noteworthy that most TOP10 variants we identified were imputed ones (92.0%), which highlights the potential of imputed WGS data for identifying new candidate variants for causal mutations. Additionally, relatively few of these TOP10 variants were annotated as intergenic variants (from 0 to 18.4%), meaning that we mostly identified either the genes or their known regulatory regions. Our results confirm the utility of using WGS data for QTL detection, although in this case our efforts were also supported by the precision of the imputation (average R^2^ of 0.84 for TOP10 variants), which was permitted by the large reference population of 1414 Holstein bulls in Run9 of the 1000 Bull Genomes Project [29].

### Limitations

The phenotypes used in this study are CH_4_ emissions predicted from milk MIR spectra, which reflect milk composition. The first consequence is that their genetic parameters may be biased compared with those of directly measured traits. Although the heritability values estimated in this study for predicted MeI and MeY are consistent with those estimated from direct measurements (0.30 and 0.33 for MeI [8, 62], 0.38 for MeY [8]), our heritability estimates for predicted MeP (0.23–0.42) are generally higher than the ones reported in the literature (0.12–0.36 [8, 11, 62–65]). This can be partly explained by the inherent heritability of milk MIR spectra. Several studies have estimated the heritability of different wavelengths in the regions of milk MIR spectra used for prediction [66–68], reaching 0.42, 0.27, and 0.30, respectively. Therefore, the traits predicted from milk MIR spectra are heritable because they are a linear combination of heritable wavelength absorbances.

The second consequence is that it was not surprising to find QTLs associated with genes that are already known to be associated with either milk composition or milk MIR spectra. This makes it difficult to verify if these QTLs are truly associated with CH_4_ production or only with milk production and composition. There can be confounding effects due to the genetic correlations between the predicted CH_4_ traits and the milk traits. We estimated genetic correlations between the six predicted CH₄ traits and milk traits using bivariate analyses with Eq. (1), that suggest moderate confounding effects with milk production and composition. Except for MeP_indirect, genetic correlations with milk yield ranged from − 0.25 to 0.23, with protein yield from − 0.18 to 0.04, and with fat yield from − 0.39 to 0.42. MeP_indirect showed stronger genetic correlations with these traits, of 0.84, 0.76, and 0.47, respectively, but presented only 2 QTLs, which contain the *DGAT1* and the *VPS13B* genes. The genetic correlations with the fat yield raises concerns about the causality of the association between the predicted CH_4_ traits and the identified genes. Further research is required to validate or invalidate these potential candidate genes. However, this would necessitate large populations with direct CH_4_ measurements to prevent the confounding effects of milk production and composition traits that are inherent to prediction from milk MIR spectra.

## Conclusions

By studying CH_4_ emissions predicted from milk MIR spectra, we were able to obtain a large amount of data, which, when combined with imputed WGS data in GWAS analyses, gave us a high detection power and a high resolution of the results. For the six CH_4_ traits examined here, we were able to identify a total of more than 100 QTLs; of these, several were shared among multiple traits, thus highlighting their common genetic basis. We identified three potential candidate genes that are involved in the production of milk FAs from VFAs (*FASN* on BTA19, *DGAT1* on BTA14, and *ACSS2* on BTA13) along with one gene that is associated with bacterial genera (*Prevotella*, *Ruminococcus*, and *Eubacterium*) with known effects on VFA and hydrogen production (*KCNIP4* on BTA6). Further research is necessary to determine if these genes are associated with predicted CH_4_ emissions or if the association is inherent to the nature of the prediction from milk MIR spectra.

## Data Availability

Raw genotype and phenotype data belong to French farmers and have commercial value. Restrictions apply to their availability, and they are not publicly available. The authors can be contacted for a reasonable request.
